# Bilateral supernumerary axillary breasts: a case report

**DOI:** 10.11604/pamj.2020.36.282.20445

**Published:** 2020-08-14

**Authors:** Khalid Mazine, Abdesslam Bouassria, Hicham Elbouhaddouti

**Affiliations:** 1 General Surgery Department, Targuist Hospital, Al-hoceima, Morocco,; 2 Department of Visceral Surgery, Hassan II University Hospital, Fes, Morocco

**Keywords:** Supernumerary breasts, mammary gland, axillary region

## Abstract

Accessory breast tissue is an uncommon condition which occurs in 0.4-6% of women. It is mostly located in the axilla and has a high incidence of being misdiagnosed. Usually it is bilateral and presents as an asymptomatic mass during pregnancy or lactation. The diagnosis of ectopic breast tissue is important as it can undergo the same pathological changes that occur in a normal breast, such as mastitis, fibrocystic disease and carcinoma. We present a case of a bilateral axillary localization of accessory breast in a 45-year-old woman. The principal symptom was pain and the clinical diagnosis was bilateral lipoma. However, subsequent imaging and histopathological examination proved it to be an accessory breast tissue.

## Introduction

Supernumerary breasts are located in 90% of cases in the thorax, 5% in the abdomen and 5% in the axillary region [1]. The diagnosis is relatively easy when it appears with nipples or milk flow, it becomes more difficult in the absence of the above indicators or in case of predominance of the fatty tissue [2], which can be quite confusing with all other etiologies of axillary masses [3]. The purpose of this observation is to specify the distinctive clinical features between a lipoma and a supernumerary breast.

## Patient and observation

**Figure 1:**
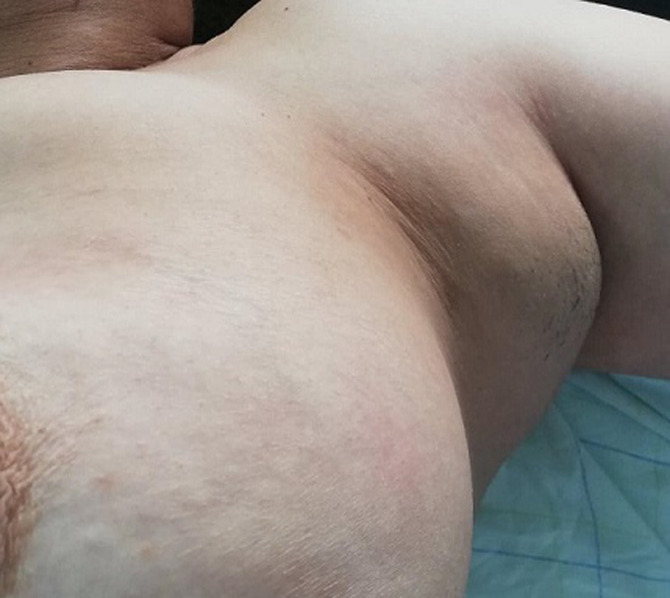
left supernumerary breast

**Figure 2:**
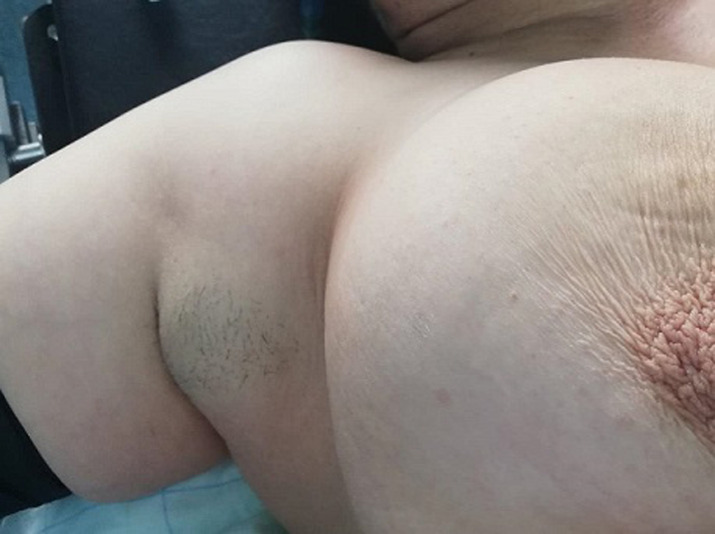
right supernumerary breast

Mrs B.K 52 years old, gravida 6, parity 5, presented with bilateral axillary masses evolving since adolescence. Initially they were small swellings of 2 cm in the right side and 4 cm in the left side, they gradually grew larger during pregnancy with a sudden, significant increase in size after delivery. They were occasionally painful and reduce the left arm movement. Physical examination revealed a 12x10 cm and 6x8 cm masses respectively in the left and right axilla they both were mobile, painless and firm in consistency. Skin over the swelling was hyperpigmented and free from underlying structures. There was no nipple or areola over the masses. The bilateral breasts were normal without any palpable axillary lymphadenopathy (Figure 1, Figure 2).

**Figure 3:**
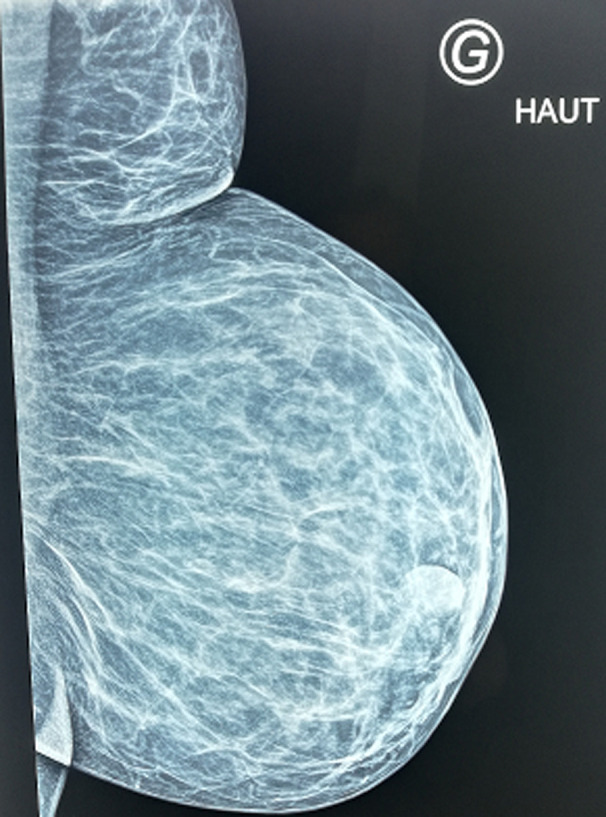
left mammography

**Figure 4:**
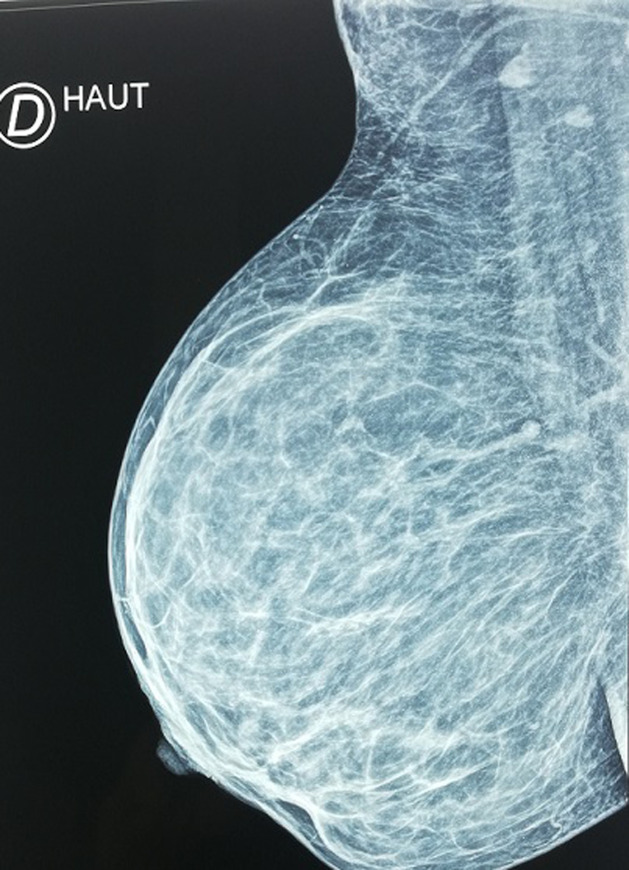
right mammography

**Investigations:** all routine investigations were within normal limits. Mammography and breast ultrasound examination gives an indication to the diagnosis of supernumerary breast tissue (Figure 3, Figure 4).

**Figure 5:**
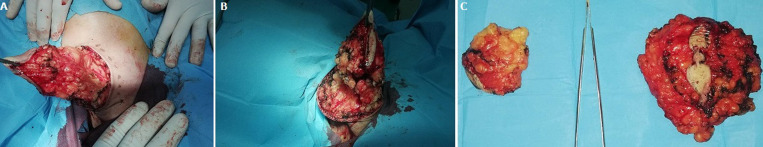
right side excision (A); left side excision (B); resected specimens (C)

**Treatment:** an excisional biopsy was performed under general anesthesia and the tissue was sent for histopathology. Grossly on sectioning, we found an adipose tissue (Figure 5). Microscopic examination showed mostly mature adipose tissue interspersed with breast tissue having lobular architecture. Numerous ducts and acini were seen lined by ductular epithelial cells. Thus, histopathological features were consistent with the accessory breast tissue.

## Discussion

Ectopic breast tissue occurs due to failure of resolution of the embryonic mammary ridge (milk line), which is an ectodermal thickening from axilla to groin bilaterally. It is the term used for both supernumerary and aberrant breast tissue, which are two distinct entities [4]. Supernumerary breasts have nipples, areolae or both with varied composition of glandular tissue [5]. They mostly present along the mammary ridge but may also occur on the cheek, neck, shoulder, thigh or buttock [6]. It is usually sporadic; however, a hereditary and familial predisposition has also been reported [7]. About 2% to 6% of females and 1% to 3% of males are affected by this condition, a third of whom have more than one area of supernumerary tissue growth. Occurrence rates vary widely on the basis of ethnicity and gender, ranging from as low as 0.6% in Caucasians to as high as 5% in Japanese females [7,8]. Supernumerary breast was categorized in 1915 by Kajava, whose classification system still remains in use today [9]. Class I consists of a complete breast including glandular tissue, nipple, and areola. Class II consists of only glandular tissue and nipple, without areola. Class III consists of only glandular tissue and areola, without nipple. Class IV consists of only glandular tissue. Class V (pseudomamma) consists of only nipple and areola, without glandular tissue. Class VI (polythelia) consists of only the nipple. Class VII (polythelia areolaris) consists of only the areola. Class VIII (polythelia pilosa) consists of only hair.

Although present at birth, it stays dormant until puberty, pregnancy, or lactation. It presents as a soft tissue swelling with or without a surrounding areola or nipple [9]. The development of breast neoplasm in ectopically located glandular tissue has been depicted. Madej *et al*. [10] describe a rare case of a 50-year-old woman who, in spite of undergoing regular mammography screening, developed an invasive accessory breast cancer. The management of ectopic breast is mainly surgical, though small size asymptomatic ectopic breasts may be managed conservatively. Excision is recommended in large size tissue for cosmetic reasons and to avoid any future complications [11]. An alternative tumescent liposuction technique was described, it allows to prevent the occurrence of the central depression appearance that is often left as a result of the adjacent fat tissue remnant following the traditional methods of resecting the accessory breast tissue [12]. This alternative approach may result in minimal scarring and better contour than can be obtained by conventional methods. Regardless of the technique used, attempts at removing accessory breast tissue can lead to surgical complications such as contour irregularities, seromas, and possibly recurrence.

## Conclusion

Supernumerary breasts are not a common entity and may sometimes pose a diagnostic challenge. Diagnosis of accessory breast tissue is important as it can undergo the same pathological changes that occur in the normal breast, such as mastitis, fibrocystic disease and even carcinoma. Surgical management is recommended for cosmetic reasons as well as to avoid any future complications.
